# How did the Good School Toolkit reduce the risk of past week physical violence from teachers to students? Qualitative findings on pathways of change in schools in Luwero, Uganda

**DOI:** 10.1016/j.socscimed.2017.03.008

**Published:** 2017-05

**Authors:** N. Kyegombe, S. Namakula, J. Mulindwa, J. Lwanyaaga, D. Naker, S. Namy, J. Nakuti, J. Parkes, L. Knight, E. Walakira, K.M. Devries

**Affiliations:** a Department of Global Health and Development, London School of Hygiene and Tropical Medicine, 15-17 Tavistock Place, London WC1H 9SH, United Kingdom; b Independent Researcher, Kampala, Uganda; c Raising Voices, 16 Tufnell Drive, Kamwokya, P.O. Box 6770, Kampala, Uganda; d University College London, Institute of Education, University College London, 20 Bedford Way, London WC1H 0AL, United Kingdom; e School of Social Sciences, Makerere University, P.O Box 7062, Kampala, Uganda

**Keywords:** Uganda, Prevention of violence against children, Corporal punishment, Positive discipline, Good School Toolkit

## Abstract

Violence against children is a serious violation of children's rights with significant impacts on current and future health and well-being. The Good School Toolkit (GST) is designed to prevent violence against children in primary schools through changing schools' operational cultures. Conducted in the Luwero District in Uganda between 2012 and 2014, findings from previous research indicate that the Toolkit reduced the odds of past week physical violence from school staff (OR = 0.40, 95%CI 0.26–0.64, p < 0.001), corresponding to a 42% reduction in risk of past week physical violence. This nested qualitative study involved 133 interviews with students, teachers, school administration, and parents, and two focus group discussion with teachers. Interviews were conducted using semi-structured tools and analysed using thematic analysis complemented by constant comparison and deviant case analysis techniques. Within a context of normative acceptance of corporal punishment this qualitative paper reports suggestive pathways related to teacher-student relationships through which reductions in violence operated. First, improved student-teacher relationships resulted in improved student voice and less fear of teachers. Second, the intervention helped schools to clarify and encourage desired behaviour amongst students through rewards and praise. Third, many teachers valued positive discipline and alternative discipline methods, including peer-to-peer discipline, as important pathways to reduced use of violence. These shifts were reflected in changes in the views, use, and context of beating. Although the GST is effective for reducing physical violence from teachers to students, violence persisted, though at significantly reduced levels, in all schools with reductions varying across schools and individuals. Much of the success of the Toolkit derives from the support it provides for fostering better student-teacher relationships and alternative discipline options. Such innovation could usefully be incorporated in teacher training syllabi to equip teachers with knowledge and skills to maintain discipline without the use of fear or physical punishment.

## Highlights

• Evaluates reduction of violence from teachers to students using Good School Toolkit.
• Reduced fear of teachers is important for students' learning and participation.
• Peer discipline valuable for improving behaviour and reducing disciplining by teachers.
• Positive discipline helps to clarify and encourage desired behaviour from students.
• Highlights potential of new norms supportive of meaningful teacher-student relationships.

## 1 Introduction

Exposure to violence in childhood is a violation of children's rights, and a risk-factor for numerous negative health and social outcomes. These include poor educational outcomes (Baker-Henningham et al., 2009, Gershoff, 2013), externalising and conduct disorders (Baker-Henningham et al., 2009, Evans et al., 2008, Holt et al., 2008, McCabe et al., 2005, Weaver et al., 2008), risky sexual behaviour (Gilbert et al., 2009), delinquency and criminal behaviour (Gilbert et al., 2009, Weaver et al., 2008), anxiety and depression (Evans et al., 2008, Holt et al., 2008, Sternberg et al., 2006); negative interpersonal conflict resolution (Breen et al., 2015); drug and alcohol misuse (Gilbert et al., 2009, Wright et al., 2013); poorer health status in adulthood (Tharp et al., 2012); and increased risk of victimisation (for girls) or perpetration (for boys) of interpersonal violence in later life (De Koker et al., 2014, Tharp et al., 2012).

Other than at home, children spend the majority of their time at school where they may be exposed to violence from fellow pupils or school staff in the form of physical, emotional or sexual violence (K. Devries et al., 2013). The UN Convention on the Rights of the Child defines corporal punishment as any intentional application of physical pain, however light, incorporating a wide range of methods including hitting (caning, beating, etc), with a hand or implement (stick, belt, etc) but can also involve kicking, shaking, pinching, burning or forced ingestion (eg washing children's mouth with soap). In addition there are other non-physical forms of punishments which are cruel and degrading and thus incompatible with the Convention, including punishment which belittles, humiliates, threatens, or scares a child (Society for Adolescent Medicine, 2003, United Nations, 1989).

Although banned in 1997 by the Ministry of Education and Sports, the prevalence of corporal punishment in Ugandan schools remains high. Data from the baseline survey of the Good School Study in the Luwero district reported students' near universal lifetime experience of violence from school staff (93% boys, 94% girls) (K. M. Devries et al., 2014). Schools in other countries in the region including Kenya and Tanzania have also been shown to have high levels of corporal punishment (United Nations Children's Fund, 2011, United Nations Children's Fund, 2012). It is only with the 2015 Children (Amendment) Act, that corporal punishment has been specifically prohibited in Ugandan schools.

In research with both teachers and students from countries including Egypt, Ghana, Sudan, and Tanzania the main reasons teachers give for their use of corporal punishment include their desire to maintain discipline in the context of perceived limited alternative discipline options (Feinstein and Mwahombela, 2010, Tao, 2015, Youssef et al., 1998), and pressure to demonstrate good academic performance alongside the belief that corporal punishment can contribute to this (Agbenyega, 2006, Elbla, 2012, Youssef et al., 1998). This is also often in the context of social norms which are permissive of corporal punishment, do not view it as inherently wrong, or normalise it through teachers’ own experience of corporal punishment in childhood (Tao, 2015, Youssef et al., 1998). In some contexts, teachers also attribute their use to stress and frustration, limited resources, and poor school environments which lack facilities such as recreational space to help mitigate unacceptable behaviours (Elbla, 2012).

**Fig. 1:**
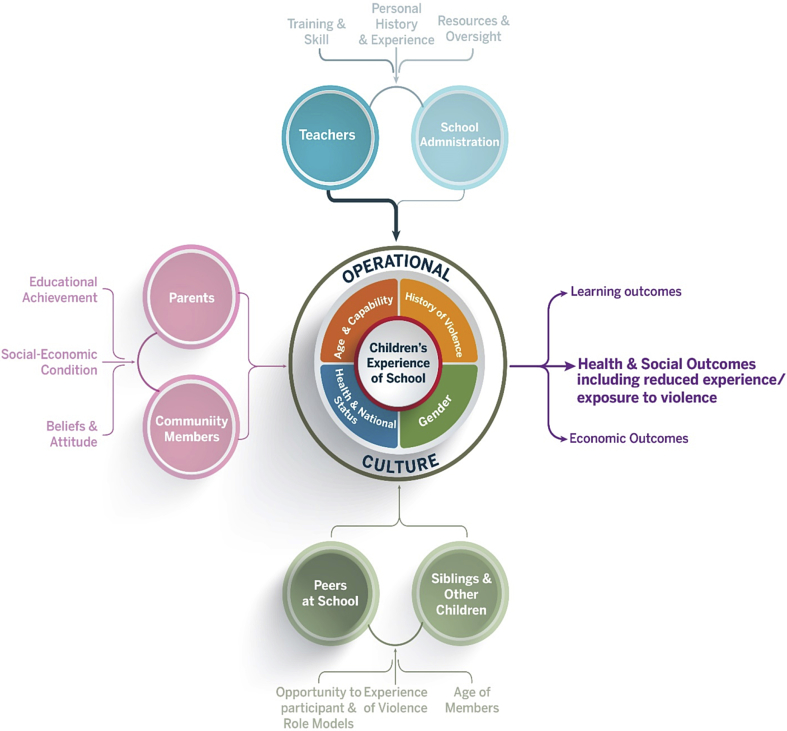
The Good School Toolkit – Theory of change.

There is a limited evidence on efficacious approaches to reduce physical violence in schools in low-and-middle-income countries (LMIC). Indeed, much of the available evidence derives from high-income countries and focuses on interventions to reduce peer violence (Farrington and Ttofi, 2009). Interventions that have been used in LMICs include Plan International's “Learn without Fear campaign” (Global Advocacy Team, 2012) and Save the Children's Violence-Free School” project in Afghanistan (Save the Children, 2010). The first intervention to be subjected to rigorous evaluation is the Good School Toolkit which is a complex behavioural intervention that draws on the Transtheoretical model (Prochaska and Velicer, 1997) and aims to improve children's experience of school through multiple entry points, as depicted in the programme Theory of Change (Fig. 1). This paper presents a qualitative evaluation of one of the entry points - teacher-student relationships.

Findings from previous research under the Good School Study, indicate that the Toolkit resulted in a 60% reduction in the odds of past week physical violence from school staff (OR = 0.40, 95%CI 0.26–0.64, p < 0.001), corresponding to a 42% reduction in risk of past week physical violence from school staff (K. M. Devries et al., 2015b). It also improved levels of school wellbeing. There was however no evidence that it impacted on educational test scores (measured using instruments validated for use in Uganda) or children's mental health (measured by the Strengths and Difficulties Questionnaire) (K. M. Devries et al., 2015b).

The aim of this paper is not to demonstrate change but rather to discuss how teachers and students interpreted and experienced the intervention, and to trace the suggestive pathways through which this translated into reduced levels of violence from teachers to students. Constructivist learning theory (CLT) offers useful theoretical grounding for understanding the pathways through which this reduction in violence occurred. Constructivism refers to the idea that people individually and socially construct meaning as they learn and engage with new ideas, concepts, knowledge and skills, and behaviour (Hein, 1991, Illeris, 2009). The significance of this paper is in developing a fuller, more nuanced understanding of school processes and relationships through which a violence prevention programme may successfully reduce corporal punishment in a low-income context in which corporal punishment is normatively accepted. It also reflects a context in which participants are required to individually and collectively construct meaning as they engage with novel ideas and concepts, some of which may be counter to their existing experience, knowledge and beliefs. Furthermore, constructivist learning theory also offers guidance on understanding barriers to learning which may limit change under complex interventions such as the Good School Toolkit. We focus on the interpersonal relationships between teachers and students and evaluate the suggestive pathways as discussed by participants. As such, this paper does not address other factors that are important for understanding the reduction in violence, including those related to the school administration, parents and community members, and children themselves (addressed in forthcoming papers).

### 1.1 The Good School Toolkit

Described in more detail in Appendix 1, the Toolkit is a school-wide intervention developed by Raising Voices and publicly available at www.raisingvoices.org. It seeks to influence the operational culture of schools through four entry points; teacher-student relationships; peer-to-peer relationships; student-and-teacher-to-school relationships; and parent-and-community-to-school governance relationships (Naker, 2017).

Guided by six core sequential steps implemented over the course of 18 months, the Toolkit includes approximately 60 activities that are conducted by school members. Activities relate to creating a better learning environment; mutual respect; understanding power relations; non-violent discipline techniques; and improving classroom management techniques. It is implemented flexibly, with no prescribed number of activities or set implementation schedule, although Raising Voices staff recommended activities considered important to each step (Naker, 2011).

Reinforcement of new information and ideas is supported by visits from Raising Voices' staff who provide direct one-on-one support to two key student and two key staff ‘protagonists’ who in turn conduct face-to-face activities with their peers, mainly in groups. Further support is provided through quarterly in-person visits to protagonists and monthly telephone calls to staff protagonists (Naker, 2011).

Elaborated in Appendix 1, schools are supported to set up various committees including a *Students' Committee*, a *Teachers' Committee,* and a *Community Committee* which co-ordinate activities and disseminate ideas introduced through each step. Schools also set up a *Student's Court* through which students are encouraged to improve behaviour through peer disciplining. *Suggestion Boxes*, *Discipline Boxes* and *Walls of Fame* are also used to increase students' voice and participation in the school (Naker, 2011).

### 1.2 The Good School Study

The Good School Study incorporated four evaluation components: a randomised controlled trial, the findings of which have been described in detail and published elsewhere (K. M. Devries et al., 2015b), a qualitative study, a process evaluation (Knight et al., 2017) and an economic evaluation (Greco et al., 2016). Key aspects of the study design are described elsewhere (K. Devries et al., 2013). The trial involved two cross sectional surveys in 42 schools and interviewed teachers and a sample of students in primary classes 5, 6 and 7 (P5, P6, P7). Baseline data were collected in 2012 and endline in 2014. Between surveys, 21 schools received the intervention while 21 were waitlisted to receive it after completion of the study. This paper reports on aspects of the qualitative study.

## 2 Methods

### 2.1 Study context

The Luwero District is located to the north-east of Kampala. In 1997 the government's Universal Primary Education (UPE) policy abolished school fees in public (UPE) schools, although parents still contribute to other educational costs (Overseas Development Institute, 2006). Children in the study setting often contribute substantially to household chores including cooking, cleaning, collecting water and firewood, farming, and tending to animals. Children also conduct chores in schools including collecting water and firewood, farming and cleaning of school buildings and compound. Some children leave home without eating because of a lack of food (K. M. Devries et al., 2014; K. M. Devries et al., 2015b). The majority of households in the district (66%) depend on subsistence farming. 18.3% live below the national poverty line as compared to a national average of 31.1% (Nadduli, 2012). Data from the study indicates that teachers feel that they are poorly remunerated with few study schools having decent staff accommodation. Many teachers describe being overworked and their schools being understaffed, many supplement their incomes with other activities including agricultural-related activities. Some teachers are food insecure.

### 2.2 Sampling and data collection

By virtue of being part of the Good School Study, all of the 42 schools included in the trial were eligible to participate in the qualitative study. Using criterion sampling (Patton, 2002) eight intervention and eight waitlisted control schools were purposively sampled for the qualitative study (from the 42 participating schools). In both groups, effort was made to maximise the heterogeneity of the sample to reflect a variety of school characteristics, contexts and experience of implementing the Toolkit (Sandelowski, 1995). Criteria upon which intervention schools were sampled were location (urban/rural), ownership (public/private), type (day/boarding), implementation experience (good progress/challenges in progress), and enthusiasm of implementation of the Toolkit activities (enthusiastic/less enthusiastic). The latter two were assessed on the experience and perception of Raising Voices staff who supported implementation. Control schools were sampled upon the same criteria (except experience and enthusiasm of implementation).

71 students, 33 teachers, eight head teachers, and 21 parents were interviewed at follow-up with roughly equal numbers of females and males included across participant groups. Two focus group discussions were also conducted with teachers. No individuals who were invited to participate declined. Interviews were conducted in Luganda or English by a team of three researchers all of whom had prior experience of researching violence. The researchers participated in five-day intensive training which included specific training on interviewing children on issues related to violence, children's rights and child protection. All interviews were conducted using a semi-structured tool and audio recorded. Interview topics were determined a priori based on a review of the literature as well as the pathways through which the Toolkit was hypothesised to operate. The tool was however sufficiently flexible to enable questions not included in the guide but of relevance to the study to be followed-up. Mean interview duration was 80 min.

Interview topics included participants' views of the school; the relationship between students and teachers; experiences of learning and teaching; relationships with peers; discipline; rewards and praise; the Good School Toolkit; and students’ experiences of corporal punishment both in-and-out of school. A total of 55 students were sampled from intervention schools. In each school (except one where we sampled six) seven students from between P5 and P7 were sampled. In each school a balance of both male and female students were sampled as well as one student who was identified by teachers to have a disability (not all schools had a child with an identified disability), one student who was considered disobedient, and one who was a member of the Good School committee or court. We also sampled children from class lists to avoid including only teacher-selected students who we suspected were amongst the most articulate, best behaved, and often from prefectural bodies. A total of 25 teachers were sampled from intervention schools. In each school we sampled three teachers (except one where we sampled four). Both male and female teachers were sampled including one staff protagonist. Some teachers were purposively sampled, for example, if students had identified them to use a lot of corporal punishment. The head teacher of each intervention school was also interviewed as well as at least two parents or caregivers (both male and female).

Student participants ranged in age from 11 to 17 years although the majority were aged between 11 and 14 years of age. Most students were from the Baganda ethnic group and were Christian by faith. Most had been attending their current school before the introduction of the Toolkit. Two were identified to have an impairment, one visual and the other auditory. Sampled teachers had taught at their schools from between nine months and 15 years. Most teachers were teaching at their school before the initiation of the Toolkit.

The primary aim of the evaluation was to assess the subjective experiences of those who had engaged with the Toolkit. The sampling was thus weighted towards them. We were keen however to examine whether pathways to change might also have been operating outside of the intervention, and therefore sampled a total of 16 students and 8 teachers from control schools. In each school one boy, one girl and one teacher were sampled, with both male and female teachers included in the sample. Individuals were identified on the advice of the teacher in charge at the time of the visit. Similar topics were examined with participants from control schools. This paper draws on the data from students and teachers from both intervention and controls schools.

### 2.3 Analysis

Data analysis was thematic and complemented by constant comparison and deviant case analysis techniques (Glaser and Strauss, 1967). The processes included regular research team meetings after each set of interviews and at completion of interviewing in each school. Meetings provided an opportunity to reflect on emerging themes, identify novel lines of enquiry, and interrogate any peculiarities in the data. Reflections on emerging themes by Raising Voices’ staff were incorporated into the on-going analysis process.

Using a single-stage transcription protocol, interviews were transcribed verbatim into English (McLellan et al., 2003). Assisted by NVIVO 10 analysis software (QSR International Pty Ltd, 2012), through constant comparison of the properties of the data a coding frame was developed which included themes identified a priori as well as those that emerged from the data. Separate coding frames were developed for intervention and control schools. Supported by the query function in NVIVO 10, charts of data summaries were developed so that comparisons could be drawn within and between cases and between intervention and control schools (Green and Thorogood, 2004, Ritchie and Spencer, 2004). This also helped to ensure that data was systematically reviewed to reduce anecdotal inclusion of data and improve transparency. Through further interrogation and comparison, concepts were further refined from which a model of the findings was developed as described below.

### 2.4 Ethical considerations

All participants provided written informed consent. During consenting processes children were informed that, based on what they disclosed, details might be passed to child protection officers. Any child protection-related referrals were based on predefined criteria that were agreed with service providers (Child et al., 2014). No referrals were made for students who participated in the qualitative study, although two were made for children whose experiences were disclosed by study participants.

Approval was provided by the ethics committees of the London School of Hygiene and Tropical Medicine and the Uganda National Council for Science and Technology (UNCST). Participants were interviewed in a private location of their choice with children interviewed within visual, although not audio, presence of others (K. M. Devries et al., 2015a; K. M. Devries et al., 2016). The study was consistent with UNICEF guidelines for safe and ethical data collection on violence against children (Graham et al., 2013). The trial is registered at clinicaltrials.gov, NCT01678846.

## 3 Results

Reductions in violence at the relationship level operated though a number of suggestive, pathways. These included improved relationships between students and teachers; encouraging desired behaviour through rewards and praise; alternative discipline options; and changes in the views, use, and context of beating.

### 3.1 Improved student-teacher relationships

The Good School Toolkit aims to support the development of new norms around how teachers can interact positively and creatively with students to foster positive teacher-student relationships (Naker, 2011). Prior to its implementation, and indeed as seen in control schools, teachers were described as the ultimate arbitrators of decisions, assessors of wrongdoing, and deliverers of discipline and punishment. In this way, little space was available for students to input into decisions that affected them and were outside of what was considered their space, for example prefectural structures. This created some distance and fear between students and teachers. Through Toolkit *suggestion boxes*, students in intervention schools described how they now had a means to tell the teachers what they thought. Many students also described how they now felt ‘more listened to’, a sentiment that extended beyond the space provided by suggestion boxes as described by a student:

“We now have a collective voice … we [are] no longer scared to ask teachers [for what we want] for fear that we will be [verbally] abused or [that the] teacher will say no.…teachers are now highly concerned to respond to us, they are no longer as tough as they were before” (Male student, rural intervention school).

Through improved relationships, students in intervention schools often described their teachers as ‘more friendly, approachable and concerned about the students’. Many students in control schools also described their teachers in positive ways, although emphasised interpersonal attributes less often. For example, they spoke positively about teachers who ‘reported to school’ ‘taught while at school or left work for them if they were absent’, and ‘marked students work’. This perhaps reflects the value students in control schools placed on the functional role of teachers and less on the quality of the relationship with them.

Improved student voice required a shift in behaviour for both teachers and students. One teacher described how:

“… giving [students] a voice [also] meant accepting for children to have a voice” (Female teacher, rural intervention school)

Which, in the context, is not something that children of this age typically have. Changes in teachers’ behaviour also affected how they exerted their power:

“What I like about this programme, is that it has created a good relationship between teachers and students … whereby students do not fear their teachers, they are open and free with us.… [before] when a child would get to school late, they would not even share their problem with the teacher, but instead get ready to receive the canes … and then go to class without having someone to listen to them. Ever since this programme started if a child comes to school late, you must find out why … then they explain … so as the teacher you get to understand the child's problem” (Female teacher, urban intervention school).

Teachers who described the importance of listening to students often also reflected on the reasons students sometimes arrived late, including being expected to conduct chores at home before being allowed to go to school.

Improved student-teacher relationships also had benefits for the class environment and student participation:

“The Good School Programme taught us that corporal punishment will not rectify the child's problem … It taught us that we should speak with the children, get to know their problem … now the student knows that the teacher isn't going to beat them. Because children fear the canes because they hurt … but once the child sees you entering in class when you don't have a cane, they're not even scared to give you a wrong answer. Currently we are better off because in the past we would enter class with canes and put it in the corner, so a student would fear to participate in class, even the one who would have gambled to give you a right answer is scared … that if they give a wrong answer they will be caned. However that is no longer the case, it has also helped us with the slow learners, they now contribute in class, because they know that even if they give you a wrong answer, you are not going to beat them” (Female teacher, urban intervention school).

One child explained how this fear had affected his learning:

“We had a teacher here and he would ask you a question, but before you could even respond he would intimidate you. He would shout saying ‘I'm going to beat you!’ so by the time he asks you a question you are already scared … you cannot give him the right answer because you are just panicking” (Male student, urban intervention school).

Teachers in intervention schools described how changes in their behaviour were also reciprocated by improvements in the behaviour of most students. In all intervention schools however, some teachers expressed a sentiment that the Toolkit:

“works for wise students but for those who are not wise, it makes them worse” (Male teacher, rural intervention school).

Teachers believed that the behaviour of a few students worsened, as the students knew they would not be beaten or receive harsh punishments in instances which would have previously attracted such. Taken as a group, students for whose behaviour the Toolkit was thought to worsen were often described as poorly disciplined, disinterested in education, and from households that did not discipline them or care about their education, as evidenced by their parents’ failure to attend school when called to discuss academic or disciplinary matters. Most students who were described in this way were in rural schools that were publicly owned. This reflected a general perception that children from private schools, and to a lesser extent, urban areas, were more invested and supported in their education.

In addition, not all students perceived all teachers to be part of the programme. They described how ‘some teachers are for Good School and some are not’, and that those who were not, continued to beat. This sentiment was also felt by a few staff protagonists who had struggled to engage some colleagues. One teacher even felt sabotaged in his efforts to conduct Toolkit activities by teachers in his school who believed he was benefiting financially from the role and were thus not willing to engage meaningfully with the intervention for example through participating in activities.

### 3.2 Recognising desired behaviour through rewards and praise

As part of positive discipline, the Toolkit encourages schools to recognise desired behaviour through rewards and praise. Teachers and students, perceived the *Wall of Fame,* to help achieve this in two main ways. First it provided clarity to students on what was considered desirable behaviour and second, through publicising this, provided motivation to students, as described by one teacher:

“At the end of term, we read out the names of students who have performed well in class so that they are recognised … It encourages them, when their friends recognise them. We even do it in classes, we tell them that you see, so and so performed well. In other words we praise them. We also pin them over there … on the wall of fame … That encourages them to study hard and make sure that their names appear. It's not only in academics, but also in other areas, like personal presentation or coming to school on time. We also write the student who has been the best behaved in the week so that students [can] read the names. [This] encourages others to strive to be recognised … students even compete to be the best in all areas, like academics, behaviour, personal presentation or even coming to school early” (Female teacher, rural intervention school).

In addition to achievements being publicly acknowledged, both students and teachers in intervention schools described how gifts were sometimes given to award achievement, although this was not part of Toolkit programming. Gifts included scholastic materials, soap, crockery and bursaries and were given for good performance in academic and extracurricular activities. In control schools, gifts were also given to acknowledge achievement, although evidence of rewards or praise being used to encourage desired behaviour was largely absent. The fact that gifts were also given in control schools suggests that rewarding achievement in this way was part of the general culture in schools in the district. The linking by participants in intervention schools of gifts to the Toolkit then appeared to be a localised interpretation or extension of the value of providing rewards and praise to encourage desired behaviours. The intensity in which rewards and praise were used varied between schools with some students in intervention schools describing neither substantive use of, nor significant change in, how students are rewarded or praised.

### 3.3 Alternative discipline options

Discipline techniques described in intervention and control schools were broadly similar. These included requiring students to conduct chores and beating. In intervention schools, unlike control schools, many teachers described changes in disciplinary techniques. They also noted that their reduced use of corporal punishment was linked to increased knowledge acquired from the intervention of alternative ways to discipline students:

“Corporal punishment was prohibited but it was still going on in schools because teachers were not given alternatives. If you want me to stop beating, what alternative should I use? That is what the Good School Programme has helped us with. It introduced positive discipline” (Female teacher, urban intervention school).

#### 3.3.1 Positive peer discipline and rules and regulations

One alternative that teachers in intervention schools thought contributed to improved discipline was the *Student Court*. Through this, students were empowered to hold each other to account against a set of rules and regulations that they had participated in developing. Many teachers valued this for its role in relieving them of having to respond to student squabbles which they had previously resolved through beating:

“We teachers are no longer bothered with the minor cases … like so and so stole my pen … The Student Court has a judge, secretary and members. They meet to discuss the offense … and bring the “criminals” and ask them to defend themselves. So the students in the Court find a way of reconciling both parties … They think of an appropriate punishment that the student has to do … they try to solve the case without coming to us teachers. We only intervene if the case is serious … or if they fail to handle it in the Court” (Female teacher, rural intervention school).

Clear rules and regulations also meant students were aware of the consequences of deviations from acceptable behaviour. Having to stand to publicly defend themselves and find witnesses to corroborate their version of events, was also something students disliked, and as such, endeavoured to ensure they were not taken to court.

#### 3.3.2 Discipline boxes

Used to write the names of those who misbehaved, Toolkit *Discipline Boxes* were valued by many teachers in intervention schools as they provided a useful deterrent for misbehaviour:

“In every class there is a discipline box … I no longer shout at the children. Instead I write their names and drop them in the box. At the end of class we open it, and we read out the names … and give them [students] a chance to suggest punishments for those who misbehaved. This has greatly helped us because students try their level best to see that their names do not appear in the discipline box. That means they have to behave well” (Female teacher, rural intervention school).

Discipline boxes were however seen by some teachers as ineffective for ‘disobedient’ children who ‘did not care whether their name was written in a box or not’ or indeed if they were beaten as a result of their names being in the box.

#### 3.3.3 Apologising

Another alternative discipline practice used in intervention schools was apologising which often took the form of apologising to their class, the school or being asked to write an apology letter. For some students being required to apologise was thought to be a worse punishment than being beaten:

“They fear so much to write an apology, and we ask them to write an apology in English, because English is a problem to them … and even sitting down and thinking about the words to include in the apology, at times they even prefer to be beaten, but we tell them that we are not going to beat you, but instead write an apology letter. Previously they knew that after committing an offence you would call them to the staff room and beat them, then they would walk away. But these days we have to counsel you, then we tell you to write an apology letter. If it was a big offence we call an assembly, and make them come to the front and ask them to kneel down and apologise to the whole school (Female teacher, urban intervention school).

While the Toolkit encourages school members to apologise as a way of taking responsibility for their actions and helping to resolve conflict, the procedure described above by the teacher reflects an extreme interpretation of the Toolkit's suggestion of the purpose of apologising.

Although many teachers recognised the value of alternative positive discipline methods, some also described them as more time consuming and disruptive to student learning:

“Positive discipline needs a lot of time to be implemented, but caning takes little time. With positive discipline you must study the children first … If a child has done something wrong and you send him out of class to do one of these alternatives, his colleagues are in class learning while he is outside for the punishment” (Male teacher, rural intervention school).

This resulted in some teachers being selective in their use of positive discipline methods. A few teachers also indicated that the greater time investment required to ensure that positive discipline was effective, meant that they used it less often with students in P7 who were under intense pressure to prepare for exams.

In intervention schools, students' views of alternative discipline methods varied. Some described a preference to be beaten ‘because it didn't hurt for long’ or ‘because it is better than having to sweep a whole compound’ which was time consuming and meant missing class or break. For some students, a ‘hybrid’ approach, in which beating was accompanied by guidance and counselling was preferred:

“I would prefer to use the guiding and counselling protocol and writing apology letters [so that students] realise their mistakes, rather than caning. You know, some teachers have a saying that ‘spare the rod and spoil the child’. You can cane but not too much. You cane a little then guide and counsel. I think those are the best ways” (Male student, urban intervention school).

Although some students in control schools also noted the value of guidance and counselling, as a group they were more accepting of beating as a disciplinary method and described non-physical, or alternative punishments, less often.

### 3.4 Changing views, use and context of beating

Although beating endured in all schools, there were notable shifts in how it was viewed and used in intervention schools.

#### 3.4.1 Views on beating

Many students in intervention and controls schools made a distinction between beating and corporal punishment. The latter often equated to the concept of ‘overbeating’ which related to the perceived fairness and proportionality of the beating. As such, some students were accepting of beating, in instances where it was meted with perceived fairness, moderation, and proportionality to the offence:

“Our teachers … use a cane fairly … where it is necessary, and don't use too much force and they also don't give us corporal punishment” (Male student, urban intervention school).

Students’ apparent acceptance of beating often reflected their internalisation of social norms around violence, given that this was a prevalent means through which they were disciplined both in-and-out of school. “Overbeating” or corporal punishment was then perceived to involve instances of beating with excessive force or resultant injury; when students were beaten on a part of the body other than the buttocks; as the only punishment for all wrong doing including minor offences; when a whole class was beaten for the misbehaviour of few; when students did not know why they were being beaten; were beaten with what was perceived as an excessive number of strokes; and when students were punished in a number of different ways for the same offence.

Through improving student-teacher relationships and alternative discipline options, beating became less frequent in intervention schools and students often described how they thought that the beating that did occur was understandable, particularly where children acted disobediently for example by refusing to follow a teacher's request. Accounts by students in intervention schools of reduced beating were also reflected in those by teachers, some of whom described how beating was ineffective and did not lead to lasting changes in students' behaviour:

“Caning is not helpful, it instils temporary fear but when the person who is feared is away, the child's behaviour does not change. If you give the child another alternative punishment [however] … they can instead reflect on what they have done wrong and they change … So we discovered that caning is not helpful” (Female teacher, urban intervention school).

These teachers contrasted however with a few who argued that what they did was not beating but instead was ‘awakening’ students to get their attention:

“For instance some children play when you are teaching. So you can cane him just to awaken him but not to hurt him … You may warn him several times, but because you want him to be attentive, you beat him but not hard, not in a bad way” (Female teacher, rural intervention school).

As such, teachers who continued to beat often described the ‘value’ of beating so long as it was ‘deserved’ and done in a controlled manner. In general, staff protagonists and those who were engaged in the intervention were less supportive of beating although some continued to beat occasionally.

#### 3.4.2 Use and context of beating

Students and teachers in intervention schools also described how the number and severity of strokes that were given as punishment reduced following the introduction of the Toolkit. Here beating was often described as a ‘last resort’ after alternative punishments, guidance and counselling were perceived to have failed. This contrasted with control schools where beating was more often described to be the dominant method of discipline:

“What I don't like is that they beat us too much. They beat you whenever you do something they don't want” (Male student, rural control school).

Some teachers in intervention schools struggled with the notion of no longer beating in any circumstances with a few describing how beating was sometimes an inevitable result of a loss of patience with a student's failure to change:

“[Beating] has advantages and disadvantages … sometimes teachers can talk about the importance of exams but don't do anything [when a child performs poorly]. If you just talk to students all the time then they get used to it and ignore you and the fact that they performed poorly and were not disciplined. That's when you find that you have lost patience and that's why I told you that I sometimes find myself having caned … and even all those who are around you think it's acceptable” (Male teacher, urban intervention school).

Some teachers in intervention schools also described parent-related challenges to their efforts to use alternative discipline methods. Though many parents viewed the intervention positively, teachers narrated how a few parents’ concern that their children were no longer beaten was so real that they withdrew their children from the school to send them to a school where students were beaten.

“Parents are resistant to change. A parent now comes and tells you if you don't beat him [son], you won't get any result out of him. The father comes very annoyed and is expecting you to support him to beat the learner. Others say if you don't beat him, I will take him away to another school where they beat. So you feel really touched and wonder what you should do. Should I cane so the kid remains here, or should I not and the kid is taken away?” (Male teacher, rural intervention school).

Differences in parents' and teachers’ approaches to disciplining was described as a particular concern as it sent mixed messages to students which some teachers felt resulted in worsening behaviour at school:

“These children are used to caning. We have stopped caning at school but the parents have not … Now the consequence is that children will become unruly. At school they are finding one thing and at home another. We should have found other solutions for helping parents to know how to discipline their children. Teachers know, but the program should have started from in communities not from the school” (Male teacher, rural intervention school).

## 4 Discussion

This paper evaluates one of the entry points through which the Good School Toolkit sought to reduce violence; the teacher-student relationship. Participants in intervention schools reported changes in both student and teacher behaviour which was described to result in improved student voice, participation, and engagement; to create more approachable teachers and to reduce students’ fear of teachers. The intervention also helped schools to clarify and encourage desired behaviour through rewards and praise, an approach that has elsewhere been found to be more efficacious than using aversive techniques (Society for Adolescent Medicine, 2003). Overall the Toolkit also supported changes in the views, use and context of beating, often with increased use of guidance and counselling. Many teachers emphasised the value they placed on positive discipline techniques, although some teachers and students perceived this to be more time consuming than beating. This might help to explain in part why violence continued in intervention schools, albeit at a reduced level (K. M. Devries et al., 2015b).

To our knowledge this study provides the first evidence of a rigorously evaluated intervention that seeks to prevent violence in schools in a LMIC context. Constructivist learning theory offers useful theoretical grounding for understanding the extent to which the intervention created change in schools. It also provides insights on barriers to learning that may result from the ‘mislearning’ of new ideas or content, or from rejection of new content. This may also help to explain why violence endured, albeit at a reduced level, in all intervention schools. Rejection may arise where content is either perceived to threaten one's pre-existing identity, or through ‘distortion’, whereby an individual may alter the content of the learning in order to be able to accommodate it within their existing beliefs and knowledge (Illeris, 2009). For teachers, the intervention often required them to unlearn some of their previous learnt behaviour of what it meant to be a teacher. This behaviour was often interpreted by students as being authoritarian leading to fear and distance from teachers. For students, the intervention provided space for their voice and participation in their school, for example through peer disciplining, but also required them to evaluate what it means to experience corporal punishment. Despite a clear stance within the Toolkit that no violence is ever justifiable, the implied acceptance of beating by some children and teachers in our sample also reflects its normative acceptance as a disciplinary method in child socialisation in schools and communities, and a rejection by some of the idea that corporal punishment is never appropriate. In many instances, teachers are seen to act *in loco parentis* and discipline a child just as a parent would had they been present, thus continuing a cycle of similar punishment as may have occurred at home (Society for Adolescent Medicine, 2003). This suggests that the use of violence within the context of controlling children's behaviour has become deeply entrenched. Changing this behaviour requires active engagement with these normative beliefs to prevent distortions of the Toolkit content while also accommodating teachers' deeply-held identity of being primarily responsible for discipline in schools and - for many - their limited knowledge of non-violent ways to pursue this. This may also be called for given that changes in violence were not universal across schools and some participants perceived the intervention to worsen student behaviour, at least initially. It is also important to note that implementation was uneven across schools and given that it is implemented flexibly by schools, not all participants received the same intensity or quality of programming (Knight et al., 2016). Furthermore, in some schools, the transferring of head teachers or teacher protagonists also impeded program activities. This highlights the importance of considering fidelity, intensity and on-going monitoring when adapting or replicating the intervention.

There is also some evidence to suggest that to accommodate the new concepts into their existing beliefs and knowledge structures, some participants may have pursued extreme interpretations of the Toolkit activities which could have resulted in unintended negative outcomes for children, for example shame and embarrassment through public apologising. While an important strength of the approach is to relinquish some control over implementation and interpretation to schools, to truly give responsibility and leadership to the school, the on-going support and mentorship by Raising Voices, and investment in rigorous research is designed to identify and addresses implementation challenges. These sessions would also be usefully complemented by feedback sessions with schools during which difficulties, challenges or unintended consequences can be openly discussed.

Changing children's experience of corporal punishment is complex as experienced by other interventions that have sought to reduce violence in schools (Global Advocacy Team, 2012, Save the Children, 2010). It requires multiple entry points and actors operating not just at inter-personal relationship levels, but also at the level of institutional structures in schools. These include the advice and support given to teachers by the administration to maintain discipline, students' participation in peer discipline (Society for Adolescent Medicine, 2003), as well as the extent to which the environment, including levels of stress and resource constraints, affect teachers' ability to internalise new learning on how to manage discipline. This is particularly so where persistence of violence norms, along with everyday stresses, may at times result in teachers losing patience and use violence to express frustration (Elbla, 2012, Global Advocacy Team, 2012, Straus, 2010, Tao, 2015).

The potential limits of normative change over an 18-month implementation period are noted, especially given that the intervention has limited influence over what happens to children outside of the school. Some participants may also need more time to go through interdependent process of individual and social learning, with time for reflection and building of trustful relationships, before demonstrating behavioural change. While there is scope to further dismantle the norm of ‘justified’ corporal punishment, the findings of this study suggest that the intervention may play an important role in starting to dismantle the normative acceptance of violence within interpersonal relationships between teachers and students. It may also reflect shifts in teachers' understanding and practice of their roles and the value they place in developing positive norms and relationships with their students. This may also provide the potential for teachers to develop a more expansive role as professionals supported by new norms that aspire toward a more meaningful relationship with children. The findings provide early evidence of fundamental change in how children are disciplined in schools. Further research on the sustainability and entrenchment of this change over time is needed.

This study has important strengths. By seeking to maximise the heterogeneity of the sampled schools, and speaking to a range of stakeholders within schools, the study reflects multiple experiences of the Toolkit and the impact that it had on relationships. This qualitative data also provides important insights for better understanding the impact of the intervention, identifying mechanisms of change, and for identifying ways in which the intervention may be improved. There is a need to provide examples for stronger guidance on dispute resolution and disciplinary practices within the student court – so that the power vested to children through this structure is not misused. The study also has limitations. All interviews were conducted at follow-up and thus provide cross-sectional data. Multiple interviews in schools throughout the process of implementation may have provided an opportunity to better examine change over the course of implementation and would have reduced the reliance on participant's recall ability. For all participants, their interview was the first time they met the researcher with many associating them with Raising Voices. Lack of trust and comfort, as well as desirability bias, may have limited participants' willingness to speak candidly about their views and experiences. Some students and teachers, for example, denied that any beating was occurring even though others reported that it was on-going. This paper is also limited by the fact that it does not discuss in detail important contextual factors that may have affected the student-teacher perspectives and relationships. These include household and community factors such as household poverty and student hunger while at school; parental views, involvement and investment in discipline practices in schools; disciplinary practices of parents which affect how students are disciplined outside of school; as well as constraints imposed by resource limitations in schools. We do however acknowledge the importance of these factors which will form the subject of forthcoming publications from the study. A fuller understanding of these factors, and the other entry-points through which the Good School Toolkit seeks to change schools' operational culture, will provide better guidance to inform future studies and interventions.

## 5 Conclusion

The Good School Toolkit is an effective intervention for reducing violence from teachers to students. Much of its success can be attributed to the support that it provides for individuals to learn new ideas, concepts and behaviours guided towards creating new norms supportive of better student-teacher relationships and to practice alternative discipline options. Training on such could usefully be incorporated in teacher training syllabi to equip teachers with knowledge and skills on how to maintain discipline in schools without fear and physical punishment while also understanding the benefits of adopting a more expansive role.
