# Supplementary material for: How did the Good School Toolkit reduce the risk of past week physical violence from teachers to students? Qualitative findings on pathways of change in schools in Luwero, Uganda
Source: Soc Sci Med. 2017 May;180:10–9. doi: 10.1016/j.socscimed.2017.03.008 (PMC5390768; doi:10.1016/j.socscimed.2017.03.008)
Supplement: Appendix [file mmc1.docx]

**Appendix 1: The Good School Toolkit**

The Good School Toolkit (GST) is publicly available at [www.raisingvoices.org](http://www.raisingvoices.org). It is designed to be implemented with minimal costs, appropriate for low-resource settings[1]. The GST is a school-wide intervention led by protagonists - two teachers, two students and two school affiliated community members who aim to influence the operational culture of the entire school through four entry points: teacher-student relationships, peer-to-peer relationships, student-and-teacher-to-school relationships and parent-and-community-to-school-governance relationships[2].

The activities are sequenced into a six step process based on the Transtheoretical model of change[3] that encourages the school to involve a wide range of stakeholders including community members, local leaders and parents.

- Step 1 aims to identify key protagonists at school and build school-wide support for the process (pre-contemplation)[2].
- Step 2 begins with baseline measurements with the aim of gathering information and views regarding where the school is starting from and helps leaders within the school to develop an outreach to parents, the local community and local officials in charge of managing the delivery and oversight of education in their area (contemplation)[2].
- Step 3 introduces a school-wide reflection on positive teacher-student relationships (preparing for action)[2].
- Step 4 introduces a school-wide reflection on how violence manifests at the school and explores practical alternatives to violent forms of discipline (action)[2].
- Step 5 provides an opportunity to reflect on what a good learning environment looks like and what role each stakeholder, including the school administration, can play in protecting all its stakeholders (maintenance of action)[2].
- Step 6 consolidates the work of the preceding steps through reflecting on what has been learned and achieved (consolidation of gains)[2].

The GST methodology enables leaders at their school to create a school-wide culture where violence is not tolerated and generates opportunities for students to participate in the decision-making processes that affect them. Through colourful and accessible learning materials, GST offers ideas for a range of activities that facilitate learning and critical reflection about violence, positive discipline, healthy relationships and gender. The overall aim is to foster egalitarian relationships and a safer overall environment within which students are likely to invest in their school, form attachments to their teachers, identify with their peers, and develop a sense of belonging[2].

**Implementation under the Good School study**

The GST contains a menu-of options/activities specific to each step. The ideal timing was to implement two steps per term, although some schools fell behind and pushed activities forward to the following term. During implementation, Raising Voices staff recommended 6 ‘critical’ activities per step that should have been consistent across schools. Beyond that, it was up to teachers and students’ committees to select which activities were most relevant to their context, and then build their action plan accordingly. Action plans were submitted and reviewed by Raising Voices staff during technical assistance visits. Some schools complemented activities with their own innovations.

The intervention is implemented through the school protagonists and Good School structures, as described below:

**Good School Committees**

Constituted under the Toolkit are three school committees namely a *Students’ Committee*, a *Teachers’ Committee,* and a *Community Committee*. Committees are responsible for co-ordinating activities, mobilising their peers and disseminating ideas introduced through each step. They also provide a forum for members to discuss their understanding and views of their experience of the Toolkit and feedback these reflections to other committees and school audiences.

**Suggestion Boxes**

Under the Toolkit, each school has a suggestion box which is designed to provide students with a medium through which to make written suggestions or feedback to the school administration about their views of the school. In most schools, suggestion boxes are opened on a weekly basis by a nominated teacher. While schools are not obliged to act on all student suggestions, the suggestion boxes provide students with a forum through which to exercise their voice.

**Class rules and regulations**

An important Toolkit activity is the collaborative development of class rules and regulations which all schools implementing the Toolkit are supported to agree. Through discussion, negotiation and agreement, classes develop a set of rules and regulations that are posted on the class wall as a reminder of the behaviour that they have agreed is unacceptable and a means to hold each other to account for avoiding this behaviour.

**Discipline Boxes**

The Toolkit recommends that discipline boxes are placed in each classroom, the purpose of which is to write down the name of any student whose behaviour contravenes pre-agreed class rules and regulations. These rules and regulations are developed and agreed by a class who also agree the associated disciplinary outcome for a particular misbehaviour. In practice, each class or school decides when to open the discipline box and to act on the contraventions of behaviour.

**Wall of Fame**

Designed to be present in a high-traffic, high-visibility area, the Wall of Fame provides an opportunities for schools to celebrate, reward and publicise students who perform well in a variety of areas including behaviour, timeliness, presentation, academic performance, helpfulness and through a positive contribution to their school. This in turn is also designed to motivate and encourage other students to exhibit the celebrated behaviours so that they can also be recognised on the Wall of Fame.

**Students’ Court**

The Student Court is a form of peer discipline through which students are provided an opportunity to hold each other to account. It is also designed to teach students about due process and to provide students with a forum through which their grievances with their peers can be heard and discussed. The Court is constituted of a judge and secretary as well as student committee members. The students are empowered to make judgement and decide on positive discipline outcomes for ‘minor’ cases. There is however some oversight of the court by teachers (in particular the teacher responsible for discipline in the school). In addition, during the Step 4 training, teacher protagonists as well as members of the student committee and court are trained in administering positive discipline, with emphasis on the following guiding principles to minimize potential for harm: fairness, relevance, proportionality, and focus on correcting behaviour (rather than punitive discipline). Some courts also use the GST booklet on Alternatives to Corporal Punishment to select appropriate forms of punishment.

In the case of a dispute between students, each is given the opportunity to give ‘their side of the story’ and is invited to bring ‘witnesses’ who can corroborate their version of events. The judge and committee, after listening to each student and consulting with the pre-agreed school rules, then make judgment on the ‘case’ and determine a positive discipline outcome for the offending student. All court decisions are also overseen by a teacher who can vacate any disciplinary action determined by the court, for example if it is considered too harsh, and suggest a more appropriate penalty or alternative. Similarly, if the Court deems a situation to be beyond its capacity, there are also mechanisms in place for the court to refer the dispute to teachers who then decide on the appropriate course of action.

**New expectations around a ‘good teacher’, ‘good student’ and good teacher-student relationships**

The GST tries to establish expectations about what a “good teacher” is – (1) a teacher who does not use corporal punishment; (2) a teacher who understands how students learn and why they may misbehave; (3) a teacher who uses creative methods of teaching; (4) a teacher who respects students and their views. These expectations are contained in a booklet and as part of a poster on “What is a Good Teacher” with these materials being used during Step 3 trainings and activities.

The GST recognizes the intrinsic value of all students and appreciates diversity of learning styles, skills, interests, aptitudes and as such, does not have an explicit part of the programme that focuses on what a ‘good student’ is. However when revising school rules (Step 5) students are asked to reflect and include expectations for student behaviour in the school (as well as expectations for teachers and parents) which creates space for schools to involve children in identifying desirable student behaviour.

The teacher-student relationship is addressed throughout the GST, and explicitly during Step 3 training. During this step, there is a session on being an effective teacher that describes the relationship teachers should have with the learners which involves aspects including involving them in lesson planning, asking them questions, and allowing them to ask teachers questions. There is also a suggested activity in Step 3 on students providing feedback to their teachers, a forum which is also supported through suggestion boxes.

1. Devries, K., et al., *The Good Schools Toolkit to prevent violence against children in Ugandan primary schools: study protocol for a cluster randomised controlled trial.* Trials, 2013. **14**(1): p. 232.

2. Naker, D., *Operational culture at schools: an overaching entry point for preventing violence against children at school*, in *For paper for Know Violence in Childhood*. 2017, Know Violence: Newark.

3. Prochaska, J.O. and W.F. Velicer, *The transtheoretical model of health behaviour change.* American Journal of Health Promotion, 1997. **12**(1): p. 38-48.
